# Potential transferability of *mcr-3* via IS*26*-mediated homologous recombination in *Escherichia coli*

**DOI:** 10.1038/s41426-018-0057-6

**Published:** 2018-04-04

**Authors:** Zheng Wang, Yulin Fu, Xiang-Dang Du, Haiyang Jiang, Yang Wang

**Affiliations:** 1 Beijing Advanced Innovation Center for Food Nutrition and Human Health, College of Veterinary Medicine, China Agricultural University, Beijing, 100193 China; 2 Beijing Key Laboratory of Detection Technology for Animal-Derived Food Safety, College of Veterinary Medicine, China Agricultural University, Beijing, 100193 China; 3 College of Veterinary Medicine, Henan Agricultural University, Zhengzhou, 450002 China

Worldwide dissemination of the mobile colistin-resistance gene *mcr-1* is of great concern to public health. Recently, a novel colistin-resistance gene, *mcr-3*, was reported to share 45.0% nucleotide sequence identity with *mcr-1*^1^. To date, *mcr-3-*positive isolates have been described in several regions (China, Denmark, and Spain) and species (*Escherichia coli*, *Salmonella Typhimurium,* and *Aeromonas veronii*)^2–4^. Although the plasmid-mediated *mcr-3* gene has been identified in most Enterobacteriaceae, with some horizontal mobile elements, such as Tn*As2* and IS*Kpn40*^1, 4, 5^, no evidence exists of *mcr-3* gene transmission mediated by these transposons or insertion sequences. Here, we describe an MCR-3-producing *E. coli* ST3634 isolate and, for the first time, identify the intermediate circle of the *mcr-3*-carrying fragment formed by a truncated insertion sequence, IS*26*, and an intact IS*15DI*, which was similar to the transfer of *mcr-1* via Tn*6330*^6^, suggesting this *mcr-3*-containing segment could be looped out via IS-mediated homologous recombination.

*E. coli* HN8 was obtained from a fecal sample of an apparently healthy pig at a conventional farm in Henan Province, China, during a routine surveillance study in 2017. PCR and sequencing analyses revealed that *mcr-3* was present in *E. coli* HN8. The *mcr-3* in HN8 shared 100% nucleotide sequence identity to the original *mcr-3* gene reported by Yin et al.^1^. Susceptibility was tested by the broth microdilution method following the CLSI guidelines, and the minimal inhibitory concentrations were interpreted from the CLSI breakpoints^7^. *E. coli* HN8 was resistant to colistin (4 mg/L), gentamicin (64 mg/L), tetracycline (64 mg/L), ampicillin (64 mg/L), florfenicol ( > 128 mg/L), and trimethoprim/sulfamethoxazole (32/608 mg/L), but susceptible to ceftriaxone (0.03 mg/L), imipenem (0.5 mg/L), aztreonam (0.03 mg/L), ciprofloxacin (0.5 mg/L), and amoxicillin–clavulanic acid (16/8 mg/L).

To further analyze this *mcr-3*-positive HN8 isolate, whole-cell DNA was extracted using a Wizard genomic DNA purification kit (Promega, Beijing, China) and used for whole-genome sequencing (WGS) on an Illumina Hiseq 2500 platform (Berry Genomics Company, Beijing, China). From the WGS data, sequences types were extracted and assigned to ST3634, and various resistance genes were detected in the WGS data, including aminoglycoside-resistance genes *aph(3′)-Ia*, *aadA5*, and *aac(3)-IId*; rifampicin-resistance gene *arr-3*; macrolide-resistance gene *mph(A)*; tetracycline-resistance genes *tet*(A) and *tet*(M); florfenicol-resistance gene *floR*, quinolone-resistance genes *qnrS1*, *qnrS2*, *oqxAB*, and *aac(6′)Ib-cr*; trimethoprim-resistance gene *drfA17*, β-lactamase-encoding genes *bla*_OXA-1_ and *bla*_TEM-1B_, tunicamycin-resistance gene *tmrB*, and bleomycin-resistance gene *ble*. Four replicons, IncX1, IncR, IncFII, and IncFIB, were detected using PlasmidFinder (https://cge.cbs.dtu.dk/services/PlasmidFinder/).

S1-PFGE and Southern blotting with an *mcr-3* probe labeled with digoxigenin revealed that the *mcr-3* gene in the HN8 isolate was located on an ~70-kb plasmid, designated pHN8 (data not shown). Although the conjugation failed by filter mating using HN8 as donors and *E. coli* J53 AzR as the recipient, we obtained the transformant *E. coli* DH5α/pHN8 by electro-transformation. S1-PFGE and PCR-based plasmid replicon typing analysis indicated the DH5α/pHN8 harbored only the IncR-type plasmid, pHN8. The IncR complex replicons were first described in the *K. pneumoniae qnrS1*-plasmid, pK245, and carry various resistance genes, including the metallo-β-lactamase genes, *bla*_VIM-1_ and *bla*_NDM-1_, in clinical Enterobacteriaceae strains^8, 9^. However, the plasmids belonging to this complex are nontransferable due to the lack of transfer elements^10^, which was consistent with our failed conjugation experiment. The transformant DH5α/pHN8 exhibited a 4- to 16-fold increase in the colistin (1 mg/L), gentamicin (32 mg/L), ampicillin (64 mg/L), and ciprofloxacin (0.5 mg/L) MIC values, compared with the recipient DH5α.

**Fig. 1:**
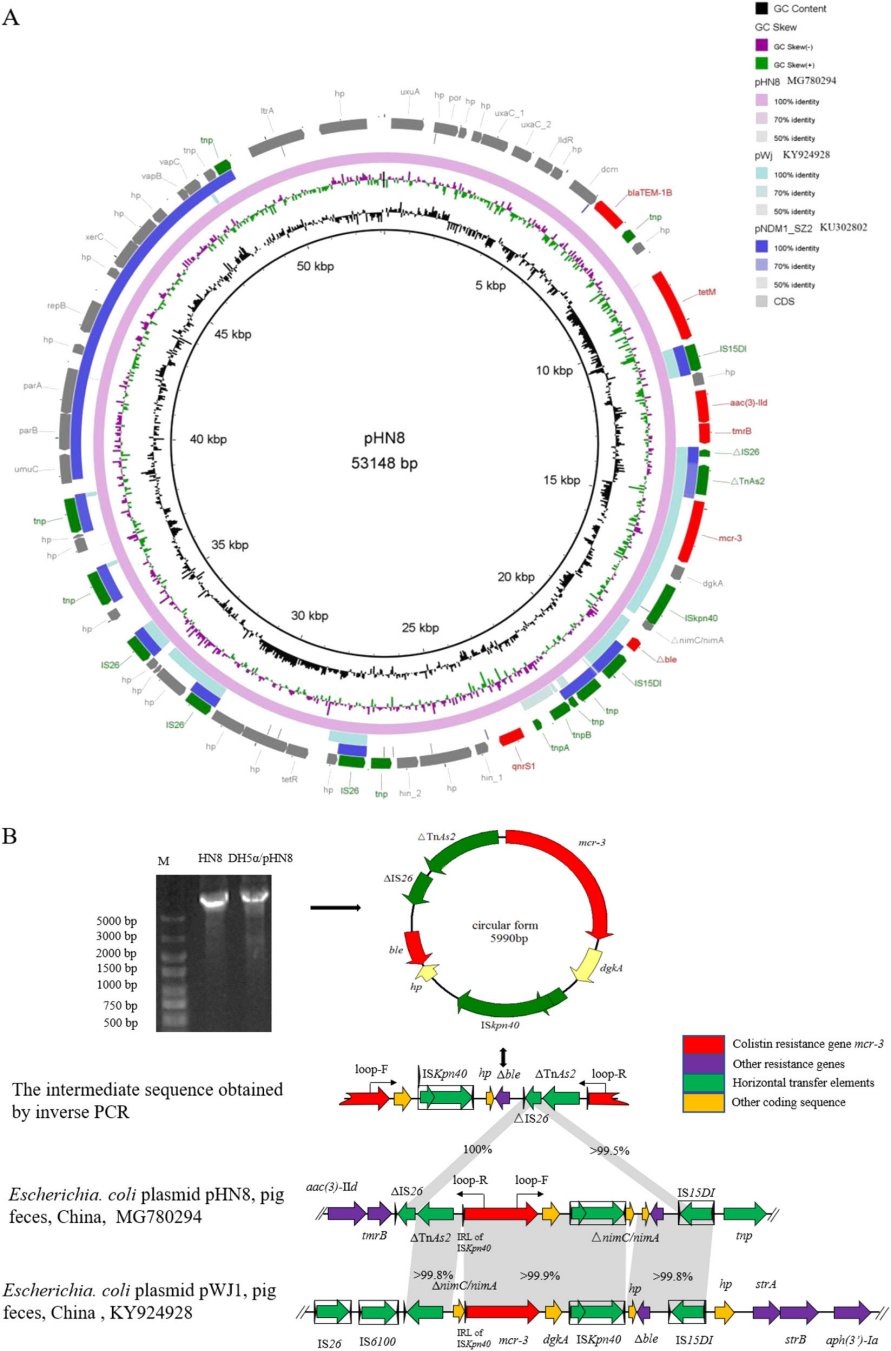
**a** Comparative analysis of pHN8 with closely related plasmids, pWJ1 and pNDM1_SZ2, using the BLAST Ring Image Generator. The concentric rings display similarity between the reference sequence in the inner ring and those in the outer rings. The outermost ring represents resistance gene (red), mobile genetic element (green), and other predicted gene (gray) coding sequences. **b** Schematic representation of the circular form obtained by sequencing the PCR product and comparing the genetic environment of the pHN8 and pWJ1 *mcr-3* genes. The gel picture of the PCR product generated by the Loop-F and Loop-R primers is shown in the top left corner. Lane M: 5000-bp DNA marker; lanes HN8 and DH5α/pHN8: inverse PCR amplicons using HN8 or DH5α/pHN8 as templates. Open reading frames (ORFs) are shown as arrows indicating the transcription direction. △ indicates a truncated gene and the box represents an intact insertion sequence

The complete pHN8 sequence was obtained by combining the Illumina Hiseq 2500 platform with single-molecule real-time sequencing (SMRT) platforms (Sinobiocore, Beijing, China). pHN8 is a 53,148-bp plasmid, consistent with the size predicted by Southern hybridization within the margin of error, with 66 open reading frames (ORFs) and an average GC content of 49.8%. It contained an 9.14-kb conservative region of the IncR-type plasmid, which shared 98.5% nucleotide sequence identity with that of *Enterobacter cloacae* plasmid pNDM1_SZ2 (GenBank accession number KU302802) and mainly harbored toxin–antitoxin system gene operon *vapB/C*, replication gene *repB*, partition protein-encoding genes *parA*/*B*, and SOS mutagenesis gene *umuC*^10^. A 32.28-kb MDR region harbored seven of the above-mentioned resistance genes (*mcr*-3, *aac(3)-IId*, *bla*_TEM-1B_, *qnrS1*, *tet*(M), *tmrB*, and *ble*), which were flanked by or interspersed with various insertion sequences, with the IS*6* family being highly abundant (IS*26*, *n* = 4, IS*15DI*, *n* = 2, Fig. 1a).

The 6288-bp *mcr-3*-carrying segment, ranging from △Tn*As2* to IS*15DI*, shared 99.9% nucleotide sequence identity to the corresponding region of the original *mcr-3*-harboring plasmid, pWJ1 (Fig. 1b). A truncated (△) IS*26* element, the 428-bp 3′-region of IS*26* (393–820 bp), was present immediately upstream of △Tn*As2*. Notably, IS*15DI*, which differed from IS*26* by only two nucleotides (A614G/A615G), was detected downstream of *mcr-3*. To determine the potential transferability of the pHN8 *mcr-3*-carrying segment, inverse PCR was performed for both HN8 and its transformant, DH5α/pHN8, using the primers located within the *mcr-3* gene (Fig. 1b). A 5990-bp circular intermediate carrying *mcr-3* flanked by △Tn*As2*, △IS*26*, and IS*Kpn40* was identified in both isolates (Fig. 1b). Based on sequence comparison of plasmid pHN8 and the 5.99-kb circular intermediate, we speculated that it was the two nearly identical insertion sequences, △IS*26* and the 3′-region of intact IS*15DI*, simultaneously contributing to the looping progress by homologous recombination (Fig. 1b). This event is consistent with previous reports in which △IS*26* and the 3′-region of intact IS*26* also formed a circular intermediate^11, 12^; however, the *mcr-3*-carrying circle mobilization evidenced in this study requires further investigation.

In summary, our study describes an MCR-3-producing *E. coli* ST3634 isolate, and *mcr-3* was located on an IncR plasmid that included a mosaic structure containing multiple IS*26* sequences. An *mcr-3*-carrying circular intermediate was mediated by a truncated IS*26* and a complete IS*15DI*, most likely via homologous recombination. Although the pHN8 belongs to an IncR-type non-conjugative plasmid, the *mcr-3* gene is transferable either to other plasmids or to chromosomes by IS-mediated transposition, and the IncR plasmid could act as a resistance gene pool in Enterobacteriaceae strains as per previous papers^8–10^.
